# Telerobotic ultrasound to provide obstetrical ultrasound services remotely during the COVID-19 pandemic

**DOI:** 10.1177/1357633X20965422

**Published:** 2022-09

**Authors:** Scott J Adams, Brent Burbridge, Leslie Chatterson, Veronica McKinney, Paul Babyn, Ivar Mendez

**Affiliations:** 1 Department of Medical Imaging, University of Saskatchewan, Canada; 2 Northern Medical Services, Department of Academic Family Medicine, University of Saskatchewan, Canada; 3 Department of Surgery, University of Saskatchewan, Canada

**Keywords:** COVID-19, robotic, telehealth, teleradiology, ultrasound, obstetrics

## Abstract

**Introduction:**

Obstetrical ultrasound imaging is critical in identifying at-risk pregnancies and informing clinical management. The coronavirus disease 2019 (COVID-19) pandemic has exacerbated challenges in accessing obstetrical ultrasound for patients in underserved rural and remote communities where this service is not available. This prospective descriptive study describes our experience of providing obstetrical ultrasound services remotely using a telerobotic ultrasound system in a northern Canadian community isolated due to a COVID-19 outbreak.

**Methods:**

A telerobotic ultrasound system was used to perform obstetrical ultrasound exams remotely in La Loche, Canada, a remote community without regular access to obstetrical ultrasound. Using a telerobotic ultrasound system, a sonographer 605 km away remotely controlled an ultrasound probe and ultrasound settings. Twenty-one exams were performed in a five-week period during a COVID-19 outbreak in the community, including limited first-, second- and third-trimester exams (*n* = 11) and complete second-trimester exams (*n* = 10). Participants were invited to complete a survey at the end of the telerobotic ultrasound exam describing their experiences with telerobotic ultrasound. Radiologists subsequently interpreted all exams and determined the adequacy of the images for diagnosis.

**Results:**

Of 11 limited obstetrical exams, radiologists indicated images were adequate in nine (81%) cases, adequate with some reservations in one (9%) case and inadequate in one (9%) case. Of 10 second-trimester complete obstetrical exams, radiologists indicated images were adequate in two (20%) cases, adequate with some reservations in three (30%) cases and inadequate in five (50%) cases. Second-trimester complete obstetrical exams were limited due to a combination of body habitus, foetal lie and telerobotic technology.

**Discussion:**

A telerobotic ultrasound system may be used to answer focused clinical questions such as foetal viability, dating and foetal presentation in a timely manner while minimising patient travel to larger centres and potential exposure to severe acute respiratory syndrome coronavirus 2 (SARS-CoV-2), during the COVID-19 pandemic.

## Introduction

The coronavirus disease 2019 (COVID-19) pandemic has exacerbated health inequities for many people around the globe.^1–3^ Challenges in accessing health-care services, including diagnostic imaging services, have been exacerbated during the pandemic, particularly in rural and remote communities where limited availability of health-care services forces patients to travel to larger centres for the care they need, increasing the risk of severe acute respiratory syndrome coronavirus 2 (SARS-CoV-2) exposure and transmission. Lack of access to care has the potential to result in substantial negative outcomes, particularly among Indigenous populations with increased health disparities and increased susceptibility to COVID-19 due to multiple factors. Virtual-care use has dramatically accelerated as a solution to promote physical distancing and to ensure that patients continue to receive the care they need, with up to a 10-fold increase in some regions.^4^ However, virtual care has mostly consisted of telephone conversations or videoconferencing between patients and their physicians.^5^ Remote solutions for diagnostic imaging are yet to be available in most communities.

Ultrasound imaging is a critical component of prenatal care to identify at-risk pregnancies and to inform clinical management, including during the COVID-19 pandemic.^6^ The International Society of Ultrasound in Obstetrics and Gynecology recommends that first-trimester dating scans and second-trimester anatomical scans continue to be performed during the COVID-19 pandemic in asymptomatic patients and COVID-19 screen-negative patients.^6^ In Saskatchewan, Canada, first- and second-trimester ultrasound exams are generally performed based on a schedule informed by the Society of Obstetricians and Gynaecologists of Canada’s clinical practice guidelines. A first-trimester ultrasound is recommended to date a pregnancy (ideally at 7 − 12 weeks’ gestation); alternatively, if menstrual dating is reliable, this can be deferred to the time of an early comprehensive pregnancy ultrasound performed at 11 − 14 weeks.^7^ A routine second-trimester ultrasound is recommended between 18 and 22 weeks to screen for foetal anomalies, number of foetuses, gestational age and the location of the placenta.^8^ Additional obstetrical ultrasound exams are guided by the patient’s clinical presentation, and current referral patterns include consultations for diagnostic ultrasound exams interpreted by radiologists to assess foetal viability, foetal presentation, amniotic fluid volume and placenta location, among other indications. These ultrasound examinations are universally available without billing directly to patients.

However, in Saskatchewan and in many communities around the world, sonographers, radiologists and obstetricians are not available on a regular basis to perform obstetrical ultrasound exams. During the COVID-19 pandemic, travel to other communities for imaging has placed prenatal patients at increased risk of exposure to SARS-CoV-2 and subsequently transmitting the virus to the community to which they return. In other communities where ultrasound exams are performed by itinerant sonographers, their travel places the community that they visit at increased risk, or places the sonographers themselves and their home communities at increased risk if travelling to an area with an outbreak. Solutions to provide local ultrasound services are urgently required in many communities around the world during the COVID-19 pandemic and beyond.

In this paper, we describe our experience using a telerobotic ultrasound system – a robotic system which allows a sonographer to perform a diagnostic ultrasound exam remotely^9^ – to perform obstetrical ultrasound exams during a COVID-19 outbreak declared in La Loche, a northern village with a population of 2372 people in Saskatchewan, Canada.^10,11^ Approximately 97% of the population of La Loche identifies as Indigenous,^12^ and it is recognised that Indigenous women have a higher rate of obstetrical complications and twofold greater maternal mortality rate than the general Canadian population.^13^ Ultrasound services in this community are normally provided by a sonographer who travels to La Loche on a chartered flight one day each month, while patients who require urgent imaging are transported to a regional hospital 507 km away or to a tertiary hospital approximately 595 km away. As La Loche experienced a COVID-19 outbreak in late April, the community was isolated, and chartered flights for ultrasound were cancelled to minimise the spread of COVID-19 to other communities and to ensure the safety of the sonographer and pilots who would be entering the community. We describe our experience providing telerobotic ultrasound services during the COVID-19 pandemic as a model for how health systems may wish to implement telerobotic ultrasound to improve access to diagnostic ultrasound imaging, increase patient safety and reduce health inequities during the pandemic and beyond.

## Methods

### Image acquisition

This prospective descriptive study was approved by the University of Saskatchewan Biomedical Research Ethics Board (Bio 15-276).

Consecutive obstetrical patients scanned using a telerobotic ultrasound system at the La Loche Health Centre between 30 April 2020 and 4 June 2020 are described in this study. Participants were invited to have a telerobotic ultrasound exam and to participate in the study if their physician or nurse practitioner requested an obstetrical ultrasound exam in La Loche. Written informed consent was obtained from each participant to have a telerobotic ultrasound exam and to have their data included in a research study. No patients invited to participate in the study declined. Patients were scheduled for telerobotic ultrasound exams based on clinical urgency indicated on the requisition.

**Figure 1.**
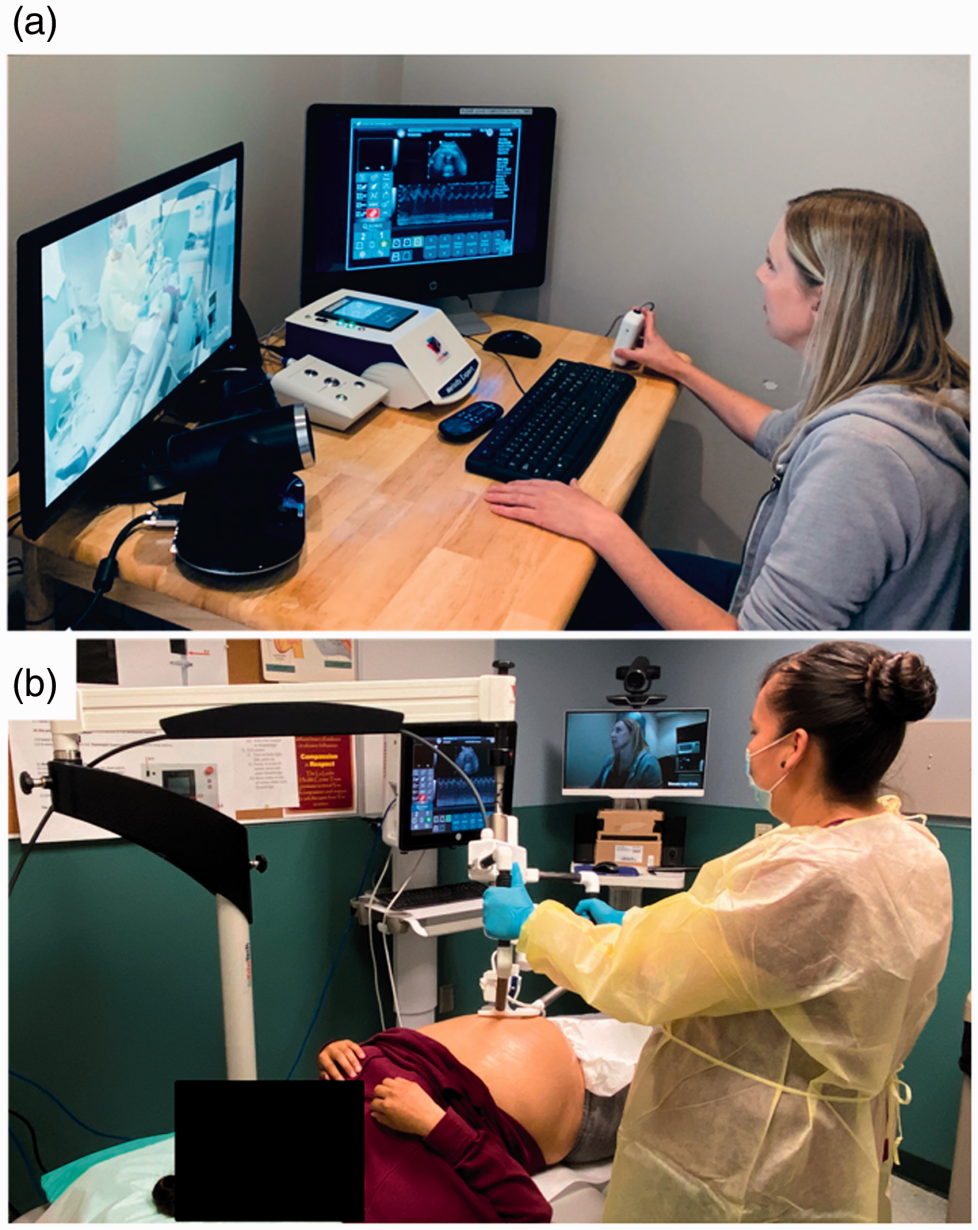
Telerobotic ultrasound system used during the coronavirus disease 2019 (COVID-19) pandemic. (a) At an ultrasound facility in Saskatoon, a sonographer manipulates a fictive ultrasound probe to control fine movements of the scanning ultrasound probe, including rotating, rocking and tilting. The ultrasound unit interface is displayed for the sonographer to view images generated in real time and to control all ultrasound unit settings remotely. A videoconferencing monitor allows the sonographer to communicate with the patient and patient-site assistant. (b) At the La Loche Health Centre 605 km away from the sonographer, an assistant positions the frame for the robotic manipulator (MELODY system) over the patient’s uterus. All the movements that the sonographer makes with the fictive probe are replicated by the ultrasound probe attached to the robotic manipulator.

Prior to each telerobotic ultrasound examination, patients were screened for COVID-19 based on provincial health authority guidelines by an assistant at the La Loche Health Centre. One of two sonographers with 13 and 16 years’ experience in ultrasound, respectively, remotely performed ultrasound examinations using a telerobotic ultrasound system (MELODY system; Société AdEchoTech, Naveil, France). The MELODY system consists of (a) a three-degrees-of-freedom robotic arm (located at the patient site) designed to manipulate an ultrasound probe and (b) a fictive probe and electronic control box (located at the sonographer site) which allows the sonographer to control the scanning ultrasound probe remotely (Figure 1).^9,14^ At the La Loche Health Centre, an ultrasound probe connected to a standard ultrasound unit (SonixTablet; Analogic, Peabody, MA) was attached to the robotic arm of the MELODY system. By manipulating a fictive probe, sonographers 605 km away from the patient at an ultrasound facility in Saskatoon, Saskatchewan, Canada, remotely controlled the ultrasound probe on the patient’s body. All fine movements of the fictive probe, including rotation, rocking and tilting, were replicated by the scanning probe in La Loche, though the translation and pressure of the probe was controlled by an assistant in La Loche who held the frame for the robotic arm. The assistant underwent a one-hour training session on how to use the MELODY system prior to assisting with patient exams, but needed no prior experience with ultrasound.

The ultrasound unit interface was transmitted to a computer monitor at the ultrasound facility in Saskatoon via Tixeo Communication Client (Tixeo, Montpellier, France). This allowed the sonographer to view ultrasound images and to control the ultrasound settings such as gain and depth remotely. The radiologist supervising each exam could also view images acquired in real time via Tixeo Communication Client. While this functionality was available for all exams and a radiologist was available if imaging findings needed to be clarified in real time as the sonographer scanned the patient, it was left to the discretion of the radiologist whether they viewed the images as they were acquired in real time or interpreted the exam based solely on the images archived in a picture archiving and communication system (PACS). A videoconferencing system (TE30 All-in-One, HD Videoconferencing Endpoint; Huawei Technologies, Shenzhen, China) was used to allow the sonographer, patient-site assistant and patient to communicate with each other via Tixeo Communication Client.^9,14^

The La Loche Health Centre and ultrasound facility in Saskatoon both had bandwidth capacity of 5 Mbps (symmetric), above the minimum requirement of 100 Kbps for robotic control data, 1 Mbps (symmetric) for videoconferencing data and 1.5 Mbps (symmetric) for ultrasound video data, as recommended by the vendor.

Sonographers performed all ultrasound exams as requested by the referring clinician based on routine imaging protocols.^8,15^ The duration of exams was determined from the time the first image was acquired to the time the last image was acquired. All images were archived in a PACS.

### Assessment

After each telerobotic ultrasound exam, patients were invited to complete a survey form to provide comments regarding their experience with the telerobotic ultrasound exam and potential advantages or disadvantages of telerobotic ultrasound during the COVID-19 pandemic. Questions included, ‘For you personally, what are the main benefits of having telerobotic ultrasound examinations performed in your community?’, ‘For you personally, what are the main disadvantages of having telerobotic ultrasound examinations performed in your community?’ and ‘Please provide any other comments about today’s experience having a telerobotic ultrasound examination’.

Following each telerobotic ultrasound exam, sonographers also completed a data-collection form, indicating technical challenges experienced during the telerobotic ultrasound exam and contributing factors limiting exam quality, including increased body habitus, foetal lie, gestational age and telerobotic technology.

Images were interpreted and reported by one of two board-certified radiologists based at the Royal University Hospital in Saskatoon. The radiologists had 6 and 30 years’ experience, respectively, in interpreting obstetrical ultrasound exams. Radiologists completed a standardised data-collection form based on Adams et al.^9^ after each study, indicating the adequacy of the images for diagnosis and whether a repeat exam was recommended due to the diagnostic quality of the exam. Determination of the adequacy of images for diagnosis was based on the principle of whether, in routine clinical practice in an outpatient clinic setting, the radiologist would ask the sonographer to acquire additional images or recommend further imaging. Diagnostic reports were generated and distributed to the referring clinician the same day or the day after each exam. The referring clinician subsequently discussed imaging findings with the patient as per routine clinical processes. In cases where images were not diagnostic, a follow-up ultrasound exam was recommended by the radiologist. The follow-up exam was provided either telerobotically or conventionally at the discretion of the referring clinician.

### Statistical and qualitative analysis

Descriptive statistics, including frequencies and proportions for categorical variables and means and standard deviations for continuous variables, were determined. Free-text responses from patient surveys were analysed using thematic analysis.^16^ This involved familiarising oneself with the data (free-text responses), generating initial codes, and searching, revising and defining themes using an approach as described by Braun et al.^16^ Two team members reviewed the free-text responses to ensure that the themes effectively represented patient responses. Data were stored on a password-protected computer, and all data was de-identified using an alternate identifier to maintain participant confidentiality.

## Results

### Patient demographics and exam indications

Twenty-one obstetrical telerobotic ultrasound exams were performed between 30 April 2020 and 4 June 2020. Three exams were follow-up studies for patients who previously had a telerobotic ultrasound exam during the study period, resulting in 18 unique patients scanned. The mean age of the patients was 28.1 years (standard deviation (*SD*) = 6.2 years).

Five first-trimester exams, 10 second-trimester complete obstetrical exams, two second-trimester limited exams and four third-trimester limited exams were performed. The mean duration of the exams was 11.4 minutes (*SD* = 7.0 minutes) for first-trimester studies, 38.1 minutes (*SD* = 6.8 minutes) for complete second-trimester exams and 17.2 minutes (*SD* = 8.7 minutes) for limited second- and third-trimester exams. No adverse events related to telerobotic ultrasound exams were reported.

Indications for first-trimester exams were dating (*n* = 3), ruling out an ectopic pregnancy (*n* = 1) and querying foetal demise (*n* = 1). Indications for second-trimester limited exams were to complete the anatomic assessment (*n* = 1) and to complete the anatomic assessment and assess foetal position (*n* = 1). Indications for third-trimester exams were to assess foetal position (*n* = 1) and to assess foetal position and growth (*n* = 1). In a further case, no previous imaging had been done, and in another, the indication was not specified.

**Figure 2.**
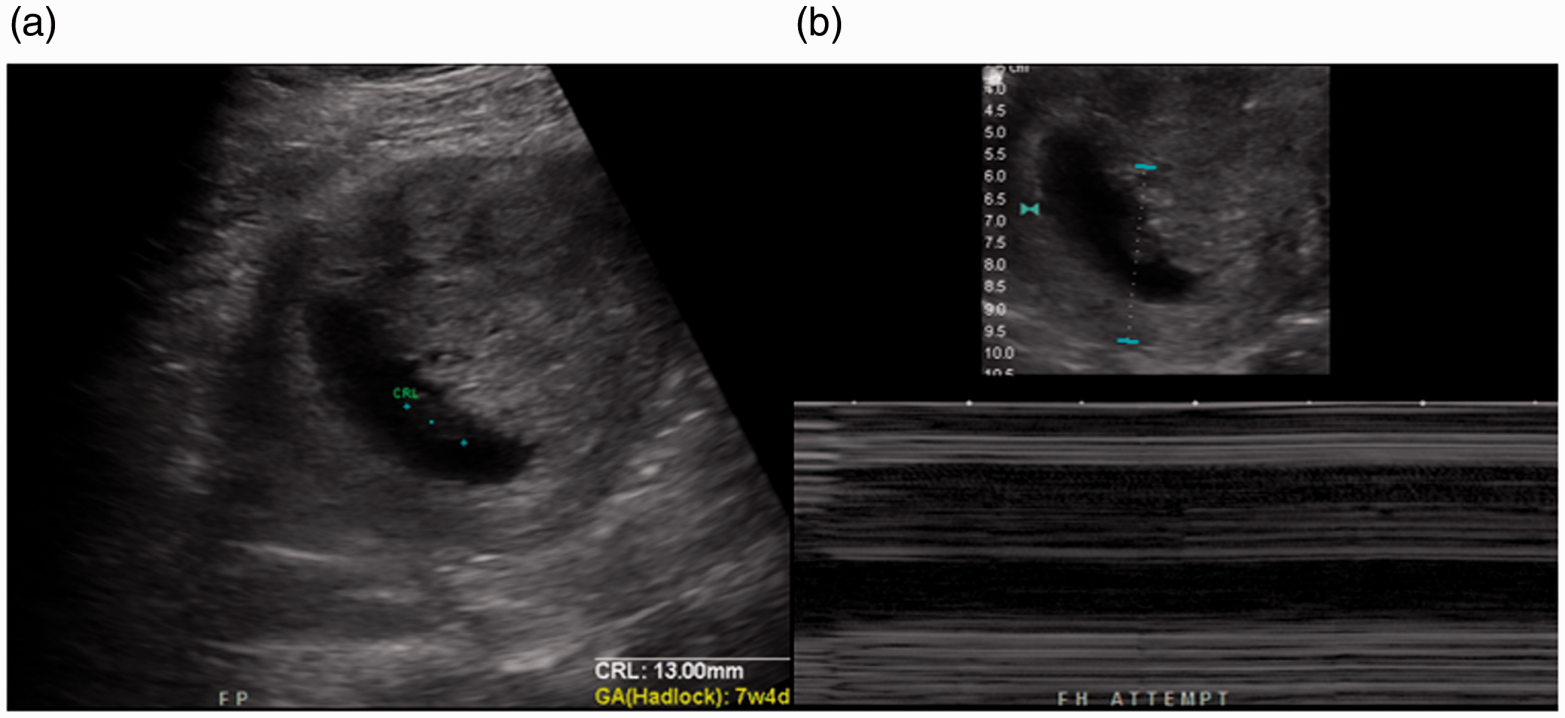
(a) Ultrasound image generated using the telerobotic ultrasound system demonstrating an embryo with a crown–rump length of 13 mm. (b) No cardiac activity is demonstrated, confirming foetal demise.

Initial telerobotic exams were repeated telerobotically for three patients: (a) a follow-up first-trimester study to confirm foetal demise (in which the follow-up exam demonstrated a crown–rump length of 13 mm and absence of cardiac activity, confirming foetal demise; Figure 2), (b) a limited second-trimester study to assess foetal presentation and (c) a second-trimester study to complete the anatomic assessment, as the assessment of some structures was suboptimal on the initial exam.

### Image assessment

For limited exams, radiologists indicated images were adequate in 9/11 (81%) cases, adequate with some reservations in 1/11 (9%) case and inadequate in 1/11 (9%) case. For the first-trimester exam where images were inadequate, the sonographer indicated the exam was limited due to body habitus, a non-distended bladder and the inability to perform endovaginal scanning.

For second-trimester complete obstetrical exams, radiologists indicated images were adequate in 2/10 (20%) cases, adequate with some reservations in 3/10 (30%) cases and inadequate in 5/10 (50%) cases.

Radiologists recommended that a follow-up study be performed for 2/11 (18%) limited studies and 7/10 (70%) second-trimester complete obstetrical studies. Of the nine examinations where a repeat study was recommended by the radiologist, seven (77%) of these exams were limited due to foetal lie, three (33%) due to body habitus and eight (88%) due to telerobotic technological limitations (with most exams having multiple contributing factors leading to suboptimal diagnostic performance, as noted by the sonographer).

### Technical challenges

Sonographers and the patient-site assistant reported that technical difficulties were experienced in 5/21 (24%) exams on four separate clinic days. In each of these cases, there was a delay between the time the mock probe was repositioned and when the ultrasound interface displayed the new corresponding image. This included an intermittent delay in ultrasound video data with no significant impact on performance of the exam (*n* = 2) and a significant delay of up to 5–10 seconds or freezing of the ultrasound video data requiring the system to be rebooted (*n* = 3). In two cases, a minimal intermittent delay continued to be experienced following rebooting.

### Patient assessment

Of 21 patients, 16 provided written comments on the survey form. Four themes related to the advantages of telerobotic sonography during the COVID-19 pandemic were identified from these comments: (a) eliminating the need to travel, (b) increased ultrasound availability, including availability for emergencies and decreased wait times for exams, (c) convenience and (d) safety, which was particularly prominent during the pandemic. Only one theme was identified related to disadvantages of telerobotic sonography during the COVID-19 pandemic: the ability to see images as they were being obtained, partially due to the positioning of the ultrasound unit in relation to the patient.

## Discussion

Obstetrical ultrasound imaging provides important information to guide clinical management by identifying at-risk pregnancies.^6^ However, the COVID-19 pandemic has increased maternal and foetal risk associated with obtaining obstetrical ultrasound due to potential exposure to SARS-CoV-2. This challenge is particularly great in geographically dispersed communities without regular access to ultrasound services, as travel to a larger centre is required in order to obtain an ultrasound exam. Previous studies have compared conventional ultrasound to telerobotic ultrasound to perform abdominal^14^ and obstetrical^9^ ultrasound exams, as well as echocardiography,^17^ generally finding excellent agreement between measurements between conventional and telerobotic scanning. In this paper, we describe use of telerobotic ultrasound as a solution for patients in underserved rural and remote communities to receive obstetrical ultrasound exams in a way that minimises travel during the COVID-19 pandemic.

Creative solutions are being explored across health-care systems to minimise exposure to SARS-CoV-2 while meeting obstetrical care needs during the COVID-19 pandemic. The International Federation of Gynecology and Obstetrics has recommended that in-person clinic visits in low-risk patients with uncomplicated pregnancies be decreased and replaced by phone calls or videoconferencing,^18^ and across specialities, there has been a dramatic increase in virtual care.^5,19,20^ However, the provision of ultrasound services is an aspect that is not served through traditional virtual-care tools.^18^ Baylor College of Medicine developed a drive-through prenatal care programme, which includes limited ultrasound exams performed from the patient’s vehicle, to reduce the number of in-person clinic visits during the COVID-19 pandemic.^21^ While this may be a promising approach in urban centres, rural and remote communities without regular access to obstetrical ultrasound exams experience unique challenges, and it is incumbent upon providers to ensure provision of diagnostic ultrasound services in a way that protects patients and health-care providers and minimises expenditure of health-care resources during the pandemic.

Patients in our study appreciated the benefits of telerobotic ultrasound as minimising the need for travel and ensuring safety, particularly important during the COVID-19 pandemic. While identifying at-risk pregnancies and providing other non-COVID-19 care continues to be of importance during the pandemic,^6^ it has also been suggested that ultrasound exams may serve as reassurance to patients and their families, which helps reduce stress and anxiety for patients and their partners during the pandemic.^6^ Obstetrical ultrasound may also help promote parental bonding with the developing foetus.^22^ As patients may otherwise travel for ultrasound imaging to a larger city alone (particularly during the COVID-19 pandemic), at a substantial distance from their home community, telerobotic ultrasound allows patients to be near their family to share their ultrasound results and to have family readily available for support in the case of negative outcomes such as foetal demise.

The benefits of telerobotic ultrasound to provide ultrasound services locally may be particularly great in Indigenous communities in Canada due to the higher rate of obstetrical complications among Indigenous peoples. A study in Quebec, Canada, found a rate of stillbirths of 5.7/1000 and 6.8/1000 births among First Nations and Inuit peoples, respectively, compared to 3.6/1000 among non-Indigenous residents.^23^ Another study in Manitoba, Canada, found a rate of stillbirth of 8.9/1000 among First Nations residents compared to 5.3/1000 among non-First Nations residents (*p* < 0.01).^24^ Higher rates of stillbirths and neonatal mortality among Indigenous populations may be due to multiple related factors, such as post-colonial policies, socio-economic status, housing, diet, tobacco and alcohol use, other environmental exposures and accessibility to health-care services.^13^ These may translate to poor foetal growth, placental disorders, congenital anomalies and diabetic and hypertensive complications, which have been shown to be strongly associated with stillbirth in First Nations and Inuit populations.^23^ Ultrasound is particularly well suited to identify resulting obstetrical complications, such as disturbances in foetal growth, amniotic fluid abnormalities or foetal anaemia.^25^ In addition to an increased rate of obstetrical complications in Indigenous populations, the arduous travel and cultural challenges experienced by many Indigenous women and families suggest that telerobotic ultrasound technology may have an important role in ensuring equitable access to ultrasound services.

Despite the many benefits of locally provided telerobotic ultrasound, some limitations to providing local ultrasound exams using telerobotic ultrasound systems should be acknowledged. The visualisation of a number of structures which are part of a second-trimester complete obstetrical exam were suboptimal on telerobotic exams due to difficulties in manipulating the probe into the correct plane using the telerobotic ultrasound system, and a repeat exam was recommended for a high proportion of complete second-trimester exams. This is consistent with our prior work, which has suggested that the foetal cavum septi pellucidi, cardiac outflow tracts, spine and kidneys are the most difficult to visualise using the telerobotic ultrasound system.^9^ Latency in ultrasound video may further contribute to difficulties in adequately assessing all required anatomy in a timely manner, and clinics must ensure sufficient bandwidth for telerobotic exams. While our results suggest that first-trimester and focused second- and third-trimester ultrasound exams can be effectively performed using a telerobotic ultrasound system, second-trimester complete ultrasound exams may be best performed through conventional (non-telerobotic) scanning. However, challenges in visualising all foetal anatomy are also common with conventional scanning, especially in obese individuals. Completion rates of a comprehensive anatomic survey are as low as 43% in normal-weight individuals and 31% in class III obese individuals, with means of 1.7 and 2.2 scans needed to complete a comprehensive anatomic survey for normal-weight individuals and for class III obese individuals, respectively.^26^

One of the disadvantages of telerobotic ultrasound, as demonstrated in previous studies, is variably longer exam times compared to conventional scanning,^14^ which is of particular concern during the COVID-19 pandemic, as the amount of time assistants are in the same room as patients should be minimised.^27^ Some authors have suggested that abbreviated ultrasound protocols can be used during the pandemic to reduce the time that the sonographer is in contact with patients.^27^ A similar justification could be used for telerobotic ultrasound to minimise contact between patients and assistants. Another strategy to reduce exam times further is capturing specific planes and completing measurements offline.^6,27^

There are several considerations to ensure patient and provider safety during telerobotic ultrasound exams during the COVID-19 pandemic. Although telerobotic ultrasound minimises potential exposure to SARS-CoV-2 among sonographers remotely performing exams, screening patients before each telerobotic ultrasound exam as per institutional protocol remains critical to ensure the safety of the assistants at the patient site and other patients who may come into contact with possible COVID-19-positive patients in common areas. Institutional guidelines and guidelines from professional societies regarding patient screening prior to ultrasound exams, including temperature checks, history regarding travel, occupation, contacts and clusters, and inquiry regarding clinical symptoms,^6,27^ should be considered when implementing a telerobotic ultrasound service. Appropriate personal protective equipment (PPE) should be worn by patient-site assistants as per institutional protocol, and consideration should be given to asking patients to wear surgical masks during exams.^28^ Similar to requirements for conventional ultrasound during the COVID-19 pandemic, the ultrasound transducer and telerobotic ultrasound unit should be cleaned with a compatible low-level disinfectant after each patient, with additional requirements following suspected or confirmed COVID-19 cases.^29^

While in this paper we demonstrate the potential for telerobotic ultrasound to facilitate non-COVID-19-related care during the pandemic, telerobotic sonography may also be used in inpatient or outpatient settings for patients who have or who are suspected to have COVID-19. Institutions have reported significantly increased ultrasound exam times for COVID-19-positive patients due to infection-control precautions (e.g. 90 minutes for a bilateral lower extremity Doppler ultrasound study to rule out deep-vein thrombosis rather than the usual 30 minutes).^27^ The use of telerobotic ultrasound would eliminate the need for sonographers to don and doff PPE to perform ultrasound exams and would minimise the use of PPE by having health-care workers already working on the COVID-19 unit assist with exams. Further, the use of telerobotic ultrasound may minimise sonographers’ potential exposure to COVID-19 and minimise possible disruptions to ultrasound operations should the sonographers need to self-isolate, particularly important considering the limited number of sonographers available in most health systems. While exam time may be longer using telerobotic ultrasound technology compared to conventional scanning, overall process time may be reduced if sonographers are not required to travel to the patient’s bedside and don and doff PPE, improving radiology throughput.

There are some study limitations. First, only telerobotic ultrasound exams were performed for each patient as part of this study, with no comparison to conventional ultrasound as a reference standard to assess diagnostic accuracy or to provide data on the proportion of exams for which follow-up would be recommended had the exams been performed conventionally. The lack of availability of ultrasound services in La Loche and the need to minimise patient and health-care provider contact during a COVID-19 outbreak in the community made it impractical to compare all telerobotic exams to conventional exams. Second, only a single reader interpreted each study, and concordance between each radiologist’s assessment regarding the diagnostic quality of each study was not assessed. This limitation is mitigated by the significant experience each radiologist has in reading obstetrical ultrasound studies, providing confidence in the interpretations provided. Further, the small sample size and the fact that all telerobotic ultrasound exams were performed at a single site limit the generalisability of the study.

## Conclusion

This study demonstrates the feasibility of telerobotic ultrasound as a means to provide obstetrical ultrasound exams during the COVID-19 pandemic in a community which would not otherwise have had locally available services due to a COVID-19 outbreak. Exams successfully answered clinical questions regarding foetal viability, dating and foetal presentation in a timely manner, though assessment of anatomy in second-trimester exams was limited due to multiple factors. Our experience provides a model for how telerobotic ultrasound may improve access to diagnostic ultrasound imaging, increase patient safety and reduce health inequities during the COVID-19 pandemic. This technology may be particularly important in Indigenous communities with increased pregnancy rates, increased rates of obstetrical complications and cultural and logistical challenges related to access to care. It is likely that the COVID-19 pandemic will further catalyse the implementation of virtual-care solutions such as telerobotic ultrasound to bring greater accessibility of health-care services, including diagnostic ultrasound, to patients. Future studies are required to determine the sustainability and clinical and economic implications of performing telerobotic ultrasound exams beyond the current COVID-19 pandemic.
